# Associations of multiple chronic disease and depressive symptoms with incident stroke among Chinese middle-aged and elderly adults: a nationwide population-based cohort study

**DOI:** 10.1186/s12877-022-03329-4

**Published:** 2022-08-12

**Authors:** Jingyang Hu, Xinyu Zheng, Guangduoji Shi, Lan Guo

**Affiliations:** 1 Department of Medical Statistics and Epidemiology, School of Public Health, Sun Yat-sen University, Guangzhou, 510080 People’s Republic of China; 2 Cerebrovascular Disease Department, Zhuhai People’s Hospital Medical Group, 519000 Zhuhai, People’s Republic of China

**Keywords:** Multiple chronic disease, Depressive symptoms, Incident stroke, Chinese, Cohort study

## Abstract

**Background:**

With the population aging, multiple chronic diseases, depressive symptoms, and stroke are increasingly common among middle-aged and elderly adults worldwide. This study aimed to explore the independent associations of multiple chronic diseases and depressive symptoms as well as their combination with incident stroke in a prospective cohort of Chinese middle-aged and elderly adults, and to sensitively estimate the association between each type of chronic disease and incident stroke.

**Methods:**

This study used data from the China Health and Retirement Longitudinal Study (CHARLS). A total of 8389 participants meeting the inclusion criteria at baseline (between 2011 and 2012) survey were included, and 7108 eligible participants completed the follow-up survey over 8 years (Wave 4, in 2018). Questionnaire information, physical examination, and clinical and biochemical measurements were collected.

**Results:**

The mean (SD) age at baseline was 58.5 (± 9.1) years. Multiple chronic disease and depressive symptoms were independently associated with incident stroke. After adjusting for control variables, patients having 1 type of chronic disease and depressive symptoms were at 1.943 (95% CI = 1.166–3.238) times higher risk of incident stroke than those without chronic disease and depressive symptoms, and patients having at least 2 types of chronic diseases and depressive symptoms were at 3.000 (95% CI = 1.846–4.877) times higher risk of incident stroke; the magnitudes of the associations increased by the numbers of having chronic diseases and depressive symptoms. Sensitivity analyses incorporating all five types of chronic disease (i.e., hypertension, dyslipidemia, heart disease, diabetes, and chronic kidney disease) showed that the magnitude of the associations between hypertension and incident stroke was most significant.

**Conclusions:**

We identified significant independent and combined longitudinal associations of multiple chronic diseases and depressive symptoms with incident stroke, and the combined associations reflected a dose–response relationship. The association between hypertension and incident stroke was strongest among the five chronic diseases.

**Supplementary Information:**

The online version contains supplementary material available at 10.1186/s12877-022-03329-4.

## Background

Stroke is one of the leading causes of death and disability globally, making stroke prevention a global health priority [1]. Although the incidence of stroke remained stable and the mortality rates for stroke decreased sharply during the past two decades [2], the number of incident strokes and the stroke-related disability-adjusted life-years [DALYs] lost and survivors are increasing [3]. Especially in low-and-middle-income countries, stroke-related mortality and DALY rates were approximately four times higher than in the high-income group [1]. A previous report in China in 2019 showed that with more than two million new cases annually, stroke is related to the highest DALYs lost of any disease [4]. With the population aging in China, the disease burden and increased risks of poor life quality for stroke in older adults are expected to increase further.

Evidence has suggested that the common chronic diseases involving hypertension, diabetes mellitus, dyslipidemia, heart diseases, and chronic kidney disease were associated with the increased risk of stroke among middle-aged and elderly adults [5, 6]. However, approximately one in three adults suffer from multiple chronic conditions globally [7], and the biological aging process may accompany the common chronic disease to increase the risk for stroke [8]. Although several effective stroke prevention strategies, such as blood pressure control, blood glucose control, or cholesterol-lowering, have been promoted [4], few studies considered the effects of the combinations of the multiple chronic diseases on incident stroke and the highest priority of the recommended prevention strategies. Moreover, the mechanism of the increased stroke risk in patients with chronic kidney disease remains unclear, with possible contributions from traditional risk factors such as diabetes and atrial fibrillation [9], and there is no consistent epidemiological evidence about whether the low estimated glomerular filtration rate (eGFR) is a risk factor of stroke independent of other cardiovascular risk factors [6]. Given that obesity was a reported risk factor for diabetes, hypertension, dyslipidemia, cardiovascular disease, and chronic kidney disease [10, 11], we should take the effects of body mass index (BMI) on the association between those mentioned above multiple chronic diseases on incident stroke into account. Moreover, some clinical and biochemical indexes, such as hemoglobin [12] or high-sensitivity C-Reactive Protein (hs-CRP) [13] have been reported to be significantly associated with the incident stroke risk. Furthermore, there is scarce study considering these clinical and biochemical indexes when estimating the associations between chronic disease and incident stroke in high-risk Chinese populations.

Except for chronic physical disease, depressive symptoms are prevalent among middle-aged and elderly adults with the cultural and social transitions recently [14] and contribute significantly to the global burden of disease [15]. A previous meta-analysis reported that the pooled prevalence of depressive symptoms among middle-aged and older adults in China was up to 22.7% [14, 16]. Previous epidemiological studies and meta-analyses have found that depressive symptoms were associated with the increased risk of stroke among middle-aged and older adults [16–18]. Moreover, depressive symptoms are common among individuals with chronic diseases, such as hypertension [19], diabetes [20], heart problems [21], dyslipidemia [22], and chronic kidney disease [23]. Although the underlying mechanisms of the associations of depressive symptoms with chronic disease and stroke are multifactorial (e.g., inflammation, endothelial dysfunction, or autonomic nerve dysfunction), it is vital to understand whether depressive symptoms play a contributor role in the associations between chronic disease and incident stroke to reduce the risk of incident stroke early.

Therefore, the objectives of this study were (1) to comprehensively test the independent associations of multiple chronic diseases and depressive symptoms as well as their combination with incident stroke in a prospective cohort of Chinese middle-aged and elderly adults (2) and to sensitively estimate the link between each type of chronic disease and incident stroke.

## Methods

### Study design and participants

**Fig. 1:**
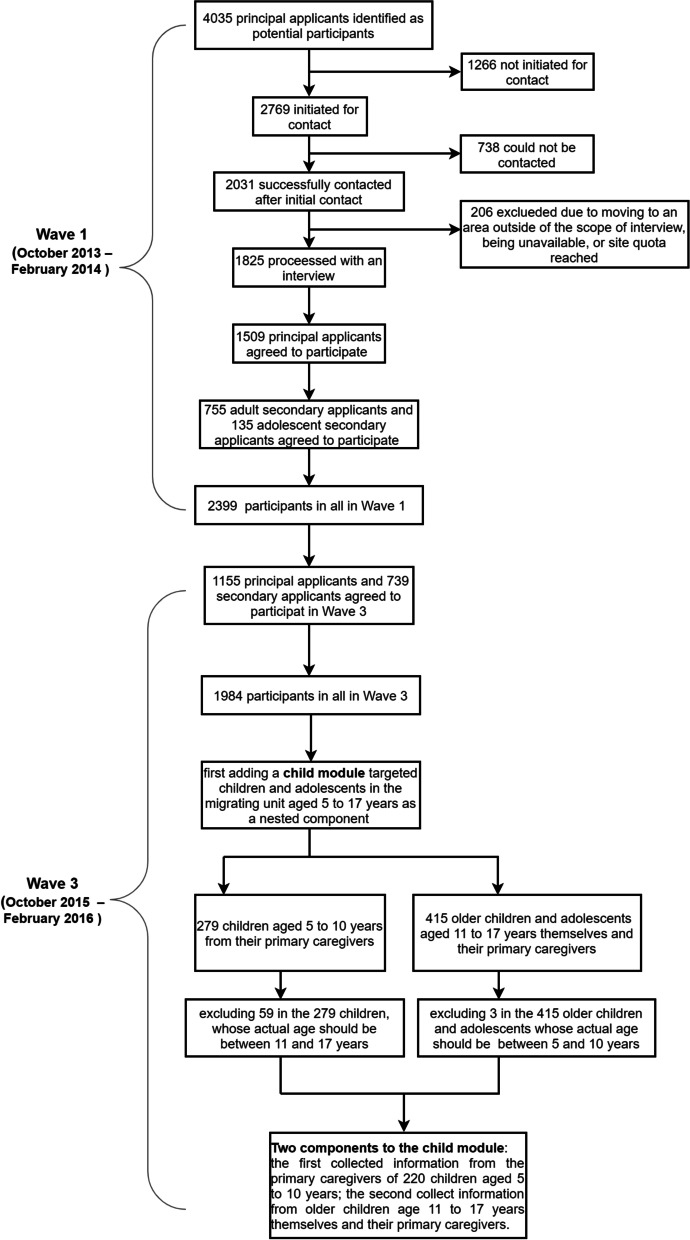
Flow chart showing the process of inclusion of the study population

Data were obtained from the China Health and Retirement Longitudinal Study (CHARLS), which is an ongoing nationally representative cohort study. Details of the study sampling method have been reported elsewhere [24]. In brief, at the baseline survey, a total of 17,708 participants were enrolled in the CHARLS from 28 of the overall provinces in China through face-to-face household interviews between 2011 and 2012, using the multistage stratified probability-proportional-to-size sampling method. All participants were followed up every two years after the baseline survey. The present study was a secondary analysis of the baseline and the Wave 4 follow-up data set of the CHARLS (Supplementary Figure S1). As shown in Fig. 1, among the recruited participants at baseline, 9627 participants providing fasting blood samples, not diagnosed with stroke before, and completing the baseline physical examination and questionnaire were selected. After excluding 216 individuals aged < 45 years, 134 having emotional/nervous or psychiatric problems, 129 having memory-related disease, 263 with brain damage or mental retardation, and 496 having vision/hearing problems or speech impediment, 8389 participants at the baseline survey were included in the current study, and 7108 eligible participants completing the follow-up physical examination and questionnaire survey over 8 years (in 2018; retention rate: 84.7%) [25].

### Data collection

#### Questionnaire

At the baseline and follow-up survey, a standard questionnaire administered by trained staff was used to collect information about sociodemographic characteristics, medical histories, and lifestyle factors, including sex (1 = male, 2 = female), age, marital status (1 = married, 2 = separated or divorced, 3 = widowed, 4 = never married), education level (1 = primary school or below, 2 = middle school, 3 = high school or above), ever smoking (1 = yes, 2 = no), last year drinking (1 = yes, 2 = no), and residence (1 = rural, 2 = urban).

Depressive symptoms were assessed by the 10-item Center for Epidemiology Scale for Depression (CESD-10) in Chinese [26], which has been validated and widely used among Chinese adults [27]. The CESD-10 consists of 10 items: (1) felt depressed, (2) bothered by little things, (3) had trouble concentrating, (4) sleep was restless, (5) everything was an effort, (6) felt hopeful, (7) felt happy, (8) felt fearful, (9) could not get going, and (10) felt lonely. The respondents were asked to rate the frequency of each item by selecting one of four response options that ranged from ‘0 = rarely or none of the time’ to ‘3 = most or all of the time’. The sum of scores ranges from 0 to 30, with higher scores indicating a higher level of depressive symptoms severity. Based on previous studies among Chinese older adults, this study adopted a validated cutoff value of 12 to classify individuals with elevated depressive symptoms (1 = yes, 2 = no) [28, 29].

#### Physical examination and anthropometric measurements

At baseline, all participants received physical examinations and anthropometric measurements conducted by well-trained examiners using standard protocols. Standing height and body weight were measured when participants were dressed in light clothes without shoes. BMI was calculated as the weight in kilograms divided by the square of height in meters (kg/m^2^). The blood pressure (including systolic and diastolic pressure) of participants was measured three times at intervals of 45 s in the left upper arm consecutively by using an automated electronic device (Omron™ HEM-7112, Omron Company, Dalian, China), and the mean values of the three measurements were used for analysis.

#### Clinical and Biochemical Measurements

Participants were asked to fast overnight, and a 4-mL sample of fasting venous blood was collected to obtain plasma and buffy coat, and another 2-mL sample of fasting venous blood was collected for glycated hemoglobin (HbA_1c_) analysis. All blood samples were stored in a local laboratory at 4 ℃ and were transported at -80 ℃ to the China Center of Disease Control (CDC) in Beijing within two weeks. Fasting blood glucose (FBG) level, HbA_1c_, lipid panel (total, HDL cholesterol, LDL cholesterol, and triglyceride), hs-CRP, creatinine, and cystatin C from frozen plasma or whole blood samples were measured. HbA_1c_ levels were measured using Boronate affinity high-performance liquid chromatography (HPLC). FBG, total cholesterol (TC), HDL cholesterol (HDL-c), LDL cholesterol (LDL-c), and triglyceride (TG) concentrations were measured using enzymatic colorimetric tests. Hs-CRP level was assessed by immunoturbidimetric assay. Creatinine concentration was assessed using the rate-blanked and compensated Jaffe creatine method. Cystatin C level was measured by using a particle-enhanced turbidimetric assay [30].

#### Measurement of chronic disease

This study considered five common chronic disease statuses: hypertension, dyslipidemia, heart disease, diabetic status, and chronic kidney disease. Hypertension was defined as self-reported history of hypertension, current use of the antihypertensive medication, systolic blood pressure ≥ 140 mmHg, or diastolic blood pressure ≥ 90 mmHg according to the 2018 Chinese hypertension guidelines [31]. Dyslipidemia was defined as self-reported history of dyslipidemia, current use of the lipid-lowering medication, TC ≥ 6.2 mmol/L, LDL-c ≥ 4.1 mmol/L, HDL-c < 1.0 mmol/L, or TG ≥ 2.3 mmol/L according to the 2016 Chinese guidelines for the management of dyslipidemia in adults [32]. Heart disease was defined as self-reported history of heart disease or currently receiving any treatment for heart disease. In this study, both prediabetes and diabetes status were taken into account. In this study, diabetic status was assessed according to the 2010 American Diabetes Association (ADA) guidelines [33]. Prediabetes was defined as an FBG level of 5.6–6.9 mmol/L or HbA_1c_ level of 5.7–6.4%; diabetes was defined as self-reported history of diabetes, current use of anti-diabetic medication, an FBG level ≥ 7.0 mmol/L, or an HbA_1c_ level ≥ 6.5%. Chronic kidney disease was defined as self-reported history of physician-diagnosed chronic kidney disease and/or eGFR < 60 mL/min/1.73 m^2^ [34]. Additionally, multiple chronic diseases were classified based on the sum categories of five chronic diseases (hypertension, dyslipidemia, heart disease, diabetes, and chronic kidney disease), and the classifications included 0 = none, 1 = one type of chronic disease, and 2 = at least two types of chronic diseases.

#### Assessment of Incident stroke events

The study outcome was incident stroke events. Incident stroke events were measured by the following question: “Have you been diagnosed or told by a doctor that you have been diagnosed with a stroke?” Participants who reported stroke during the follow-up period were defined as having an incident stroke, and the date of stroke diagnosis was recorded as being between the date of the last review and that of the interview reporting an incident stroke [35, 36].

### Statistical analysis

We calculated that with a two-sided α of 0·05 and 80% power, 2356 participants were required to show a significant association between dyslipidemia and incident stroke, with an HR of 2.4; 2601 participants were required to detect a significant association between hypertension and incident stroke, with an hazard ratio (HR) of 2.20; 3503 participants were required to show a significant association between diabetes mellitus and incident stroke, with an HR of 2.17; 4770 participants were required to detect a significant association between heart problems and incident stroke, with an HR of 1.78; 6018 participants were required to a significant association between chronic kidney disease and incident stroke, with an HR of 1.76 [37–40]. Sample size and post-hoc power were estimated using PS software (version 3.0.2). Descriptive analyses were used to describe the sample characteristics, and the data are described as number (%), mean (standard deviation, SD), or median (interquartile range, IQR) as appropriate. Baseline characteristics are summarized according to depressive symptoms and compared between participants with and without elevated depressive symptoms using the Chi-square test for categorical variables and the t-test or Wilcoxon test for continuous variables. The person-time of follow-up for each participant was calculated from the date of the baseline survey to the date of the stroke diagnosis, loss to follow-up, death, or the end of follow-up, whichever came first. Incidence rates of stroke events per 1000 person-years were calculated. The cumulative hazards of incident stroke by chronic diseases using the unadjusted Nelson-Aalen curves with log-rank tests were estimated. Cox proportional hazards models were performed to investigate the independent associations of multiple chronic disease status and depressive symptoms as well as their combination with incident stroke, and hazard ratios (HRs) with 95% confidence intervals (CIs) were calculated. Three models were estimated: in model 1: the unadjusted model; in model 2, gender, age, marital status, education level, ever smoking, last year drinking, residence, BMI, and depressive symptoms scores at baseline were adjusted; in model 3, adjusting for the variables in model 2 plus clinical variables associated with incident stroke in the univariable analyses. Moreover, multiplicative interaction was tested by incorporating a cross-product term for multiple chronic diseases and depressive symptoms along with the main effect terms for each to the multivariable Cox proportional hazards models. Sensitivity analyses were performed to incorporate all the five types of chronic disease in the models to test their associations with incident stroke. Observations with missing data (less than 0.1) were eliminated in the Cox proportional hazards models. We have tested the proportional hazards assumption for the Cox models using Schoenfeld’s residuals, and the proportional hazards assumption was upheld throughout (*P* > 0.05). All statistical analyses were conducted using Stata 16.0 SE (StataCorp, College Station, Texas, USA). All statistical tests were two-sided, and a *P* value of less than 0.05 was considered statistically significant.

## Results

### Baseline characteristics of participants according to depressive symptoms status

Table 1 summarizes the baseline characteristics of the 8389 participants. The mean (SD) age at baseline was 58.5 (± 9.1) years, and 4478 (53.4%) were females. A total of 1569 (18.7%) participants reported elevated depressive symptoms (CES-D scores ≥ 12). Univariate analyses showed that participants with elevated depressive symptoms were more probably to be female (65.3% vs. 50.5%, *P* < 0.001), older (59.9 [± 9.0] years vs. 58.1 [± 9.0] years, *P* < 0.001), be separated or divorced (2.1% vs. 0.7%, *P* < 0.001), be with primary school or below education level (79.7% vs. 62.9%, *P* < 0.001), be never smokers (67.5% vs. 60.2%, *P* < 0.001), be not current drinkers (80.3% vs. 72.7%, *P* < 0.001), live in rural area (94.2% vs. 89.7%, *P* < 0.001), have a history of hypertension (39.6% vs. 36.9%, *P* < 0.05), have a history of heart disease (17.5% vs. 9.6%, *P* < 0.001), and have a history of chronic kidney disease (9.9% vs. 5.1%, *P* < 0.001). Moreover, the differences between those with and without depressive symptoms were statistically significant in the distribution of BMI, diastolic pressure, creatine, TC, TG, HDL-c, LDL-c, hemoglobin, and uric acid (*P* < 0.05).

**Table 1:** Baseline characteristics of participants according to depressive symptoms status

**Variables**	**Baseline survey (*n* = 8,389)**	**Depressive symptoms, n (%)**		*P*-value^#^
		**Yes (*n* = 1659)**	**No (*n* = 6730)**	
**Gender**				
Male	3905 (46.5)	575 (34.7)	3330 (49.5)	< 0.001
Female	4478 (53.4)	1083 (65.3)	3395 (50.5)	
Missing data	6 (0.1)			
**Age**, mean (SD), year	58.5 (9.1)	59.9 (9.0)	58.1 (9.0)	< 0.001
**Marital status**				
Married	7521 (89.7)	1372 (82.7)	6149 (91.4)	< 0.001
Separated or divorced	81 (1.0)	35 (2.1)	46 (0.7)	
Widowed	745 (8.9)	239 (14.4)	506 (7.5)	
Never married	42 (0.5)	13 (0.8)	29 (0.4)	
**Education level**				
Primary school or below	5554 (66.2)	1322 (79.7)	4232 (62.9)	< 0.001
Middle school	1814 (21.6)	246 (14.8)	1568 (23.3)	
High school or above	1015 (12.1)	91 (5.5)	924 (13.7)	
Missing data	6 (0.1)			
**Ever smoking**				
No	5169 (61.6)	1120 (67.5)	4049 (60.2)	< 0.001
Yes	3220 (38.4)	539 (32.5)	2681 (39.8)	
**Last year drinking**				
No	6223 (74.2)	1332 (80.3)	4891 (72.7)	< 0.001
Yes	2166 (25.8)	327 (19.7)	1839 (27.3)	
**Residence**				
Rural	7601 (90.6)	1563 (94.2)	6038 (89.7)	< 0.001
Urban	787 (9.4)	96 (5.8)	691 (10.3)	
Missing data	1 (0)			
**BMI,** mean (SD), kg/m^2^	23.73 (4.45)	23.25 (4.16)	23.85 (4.51)	< 0.001
**Systolic pressure**, median (IQR), mmHg	126.67 (114.76, 141.33)	125.67 (113.67, 141.67)	127.00 (115.00, 141.33)	0.115
**Diastolic pressure**, median (IQR), mmHg	75.00 (67.33, 83.33)	74.33 (66.67, 82.67)	75.00 (67.67, 83.33)	0.040
**hs-CRP**, median (IQR), mg/dL	1.04 (0.54, 2.17)	1.02 (0.54, 2.31)	1.04 (0.55, 2.14)	0.874
**FBG**, mean (SD), mmol/L	6.11 (1.94)	6.05 (1.88)	6.13 (1.96)	0.144
**HbA**_**1c**_**,** mean (SD) (%)	5.28 (0.82)	5.31 (0.87)	5.27 (0.81)	0.078
**Serum creatinine**, mean (SD), μmol/L	69.05 (21.33)	65.91 (15.81)	69.83 (22.42)	< 0.001
**Total Cholesterol**, mean (SD), mmol/L	5.02 (1.01)	5.07 (1.00)	5.01 (1.01)	0.035
**Triglyceride**, mean (SD), mmol/L	1.50 (1.21)	1.42 (1.03)	1.52 (1.25)	0.005
**HDL-c**, mean (SD), mmol/L	1.32 (0.40)	1.36 (0.41)	1.31 (0.39)	< 0.001
**LDL-c**, mean (SD), mmol/L	3.04 (0.90)	3.10 (0.90)	3.03 (0.90)	0.005
**Hemoglobin**, mean (SD), g/dL	14.43 (2.18)	14.20 (2.17)	14.48 (2.18)	< 0.001
**Uric Acid**, mean (SD), μmol/L	265.46 (75.17)	252.62 (72.76)	268.63 (75.42)	< 0.001
**Cystatin C,** mean (SD), mg/L	1.01 (0.28)	1.02 (0.26)	1.01 (0.29)	0.119
**Hypertension**				
No	5249 (62.6)	1002 (60.4)	4247 (63.1)	0.041
Yes	3140 (37.4)	657 (39.6)	2483 (36.9)	
**Dyslipidemia**				
No	5885 (70.2)	1176 (70.9)	4709 (70.0)	0.465
Yes	2504 (29.8)	483 (29.1)	2021 (30.0)	
**Heart problems**				
No	7451 (88.8)	1369 (82.5)	6082 (90.4)	< 0.001
Yes	938 (11.2)	290 (17.5)	648 (9.6)	
**Diabetic Status**				
Others	3494 (41.6)	722 (43.5)	2772 (41.2)	0.225
Prediabetes	3783 (45.1)	725 (43.7)	3058 (45.4)	
Diabetes	1112 (13.3)	212 (12.8)	900 (13.4)	
**Chronic kidney disease**				
No	7880 (93.9)	1495 (90.1)	6385 (94.9)	< 0.001
Yes	509 (6.1)	164 (9.9)	345 (5.1)	
**CESD-10 scores, median (IQR)**	7.00 (3.00, 11.00)	16.00 (14.00, 20.00)	6.00 (3.00, 8.00)	< 0.001

*Abbreviation*: *SD* Standard deviation, *IQR* Interquartile range, *hs-CRP* High sensitivity C-reactive protein, *FBG* Fasting blood glucose, *HDL-c* HDL cholesterol, *LDL-c* LDL cholesterol, *BMI* Body mass index, *CESD-10* The 10-item Center for Epidemiology Scale for Depression (CESD-10)

^#^ The Chi-square test for categorical variables and the t-test or Wilcoxon test for continuous variables were used to assess the differences between the groups

### Baseline characteristics of participants according to incident stroke status

A total of 409 participants aged 45 years or older experienced incident stroke during the follow-up period, and the incidence rate of stroke was 7.47 per 1000 person-years. The mean (SD) follow-up time was 7.70 (± 1.31) years. Table 2 shows that without adjusting for other variables, older age (HR = 1.040, 95% CI = 1.030–1.050), being widowed (HR = 1.675, 95% CI = 1.260–2.225), higher BMI (HR = 1.044, 95% CI = 1.025–1.064), higher systolic pressure or diastolic pressure, having a history of hypertension (HR = 2.469, 95% CI = 2.030–3.004), having a history of dyslipidemia (HR = 1.776, 95% CI = 1.457–2.165), having a history of heart disease (HR = 1.807, 95% CI = 1.401–2.330), having a history of prediabetes (HR = 1.273, 95% CI = 1.034–1.568) or diabetes (HR = 1.399, 95% CI = 1.009–1.939), and having higher CES-D scores (HR = 1.024, 95% CI = 1.009–1.039) were positively associated with incident stroke. Moreover, regarding the clinical and biochemical variables, a higher level of hs-CRP, FBG, creatine, TC, TG, LDL-c, hemoglobin, uric acid, and cystatin C was positively associated with incident stroke. In comparison, a higher level of HDL-c was negatively associated with incident stroke.

**Table 2:** Baseline characteristics of participants according to incident stroke status

**Baseline characteristics**	**Participants, 8-year follow up, n (%)**			***P*-value**^**#**^	**Unadjusted model**	
	**Total (*n* = 7108)**	**Stroke**				
		**Yes (*n* = 409)**	**No (*n* = 6699)**		**HR (95% CI)**	***P*-value**
**Gender**						
Male	3172 (44.6)	197 (6.2)	2975 (93.8)	0.141	1.00 (reference)	
Female	3931 (55.3)	212 (5.4)	3719 (94.6)		0.865 (0.712–1.050)	0.142
Missing data	5 (0.1)					
**Age**, median (IQR), year	57.0 (51.0, 64.0)	60.0 (56.0, 67.0)	57.0 (50.0, 63.0)	< 0.001	1.040 (1.030–1.050)	< 0.001
**Age group**, year						
≥ 45	329 (4.6)	3 (0.9)	326 (99.1)	< 0.001	1.00 (reference)	
45–60	4198 (59.1)	202 (4.8)	3996 (95.2)		5.393 (1.725–16.862)	0.004
60–70	1881 (26.5)	141 (7.5)	1740 (92.5)		8.485 (2.704–26.624)	< 0.001
> 70	689 (9.7)	62 (9.0)	627 (91.0)		10.238 (3.214–32.613)	< 0.001
Missing data	11 (0.2)					
**Marital status**						
Married	6399 (90.0)	352 (5.5)	6047 (94.5)	0.001	1.00 (reference)	
Separated or divorced	61 (0.9)	1 (1.6)	60 (98.4)		0.293 (0.041–2.086)	0.220
Widowed	606 (8.5)	55 (9.1)	551 (90.9)		1.675 (1.260–2.225)	< 0.001
Never married	42 (0.6)	1 (2.4)	41 (97.6)		0.424 (0.060–3.021)	0.392
**Education level**						
Primary school or below	4837 (68.1)	292 (6.0)	4545 (94.0)	0.153	1.00 (reference)	
Middle school	1506 (21.2)	71 (4.7)	1435 (95.3)		0.779 (0.601–1.010)	0.059
High school or above	763 (10.7)	45 (5.9)	718 (94.1)		0.976 (0.713–1.336)	0.880
Missing data	2 (0)					
**Ever smoking**						
No	4441 (62.5)	239 (5.4)	4202 (94.6)	0.082	1.00 (reference)	
Yes	2667 (37.5)	170 (6.4)	2497 (93.6)		1.189 (0.977–1.448)	0.084
**Last year drinking**						
No	4757 (66.9)	274 (5.8)	4483 (94.2)	0.976	1.00 (reference)	
Yes	2351 (33.1)	135 (5.7)	2216 (94.3)		0.993 (0.808–1.220)	0.948
**Residence**						
Rural	6589 (92.7)	389 (5.9)	6200 (94.1)	0.055	1.00 (reference)	
Urban	518 (7.3)	20 (3.9)	498 (96.1)		0.645 (0.411–1.011)	0.056
Missing data	1 (0)					
**BMI,** mean (SD), kg/m^2^	23.6 (4.1)	23.5 (4.1)	24.5 (4.3)	< 0.001	1.044 (1.025–1.064)	< 0.001
**Systolic pressure**, median (IQR), mmHg	126.0 (114.0, 140.3)	134.3 (120.8, 150.5)	125.3 (113.7, 139.7)	< 0.001	1.008 (1.005–1.010)	< 0.001
**Diastolic pressure**, median (IQR), mmHg	74.3 (67.0, 83.0)	78.3 (69.8, 87.2)	78.3 (69.8, 87.2)	< 0.001	1.027 (1.019–1.036)	< 0.001
**hs-CRP**, median (IQR), mg/dL	0.97 (0.53, 1.97)	1.25 (0.64, 2.48)	0.95 (0.52, 1.94)	< 0.001	1.014 (1.006–1.022)	< 0.001
**FBG**, mean (SD), mmol/L	5.88 (1.45)	6.08 (1.68)	5.87 (1.43)	0.006	1.071 (1.021–1.123)	0.005
**HbA**_**1c**_**,** mean (SD) (%)	5.19 (0.61)	5.24 (0.56)	5.18 (0.61)	0.069	1.132 (0.989–1.296)	0.072
**Serum creatinine**, mean (SD), μmol/L	67.81 (15.92)	69.55 (16.86)	67.71 (15.85)	0.024	1.007 (1.001–1.012)	0.023
**Total Cholesterol**, mean (SD), mmol/L	5.01 (1.00)	5.15 (0.98)	5.00 (1.00)	0.004	1.141 (1.042–1.248)	0.004
**Triglyceride**, mean (SD), mmol/L	1.46 (1.18)	1.57 (1.00)	1.45 (1.20)	0.045	1.065 (1.001–1.134)	0.047
**HDL-c**, mean (SD), mmol/L	1.33 (0.39)	1.28 (0.39)	1.34 (0.39)	0.005	0.688 (0.529–0.893)	0.005
**LDL-c**, mean (SD), mmol/L	3.04 (0.89)	3.17 (0.92)	3.03 (0.89)	0.003	1.173 (1.057–1.302)	0.003
**Hemoglobin**, mean (SD), g/dL	14.41 (2.19)	14.65 (2.37)	14.39 (2.18)	0.020	1.050 (1.007–1.095)	0.021
**Uric Acid**, mean (SD), μmol/L	260.66 (71.51)	267.84 (77.33)	260.22 (71.12)	0.038	1.001 (1.000–1.003)	0.042
**Cystatin C,** mean (SD), mg/L	0.99 (0.23)	1.03 (0.24)	0.99 (0.23)	0.003	1.667 (1.220–2.277)	0.001
**Hypertension**						
No	4551 (64.0)	174 (3.8)	4377 (96.2)	< 0.001	1.00 (reference)	
Yes	2557 (36.0)	235 (9.2)	2322 (90.8)		2.469 (2.030–3.004)	< 0.001
**Dyslipidemia**						
No	5149 (72.4)	246 (4.8)	4903 (95.2)	< 0.001	1.00 (reference)	
Yes	1959 (27.6)	163 (8.3)	1796 (91.7)		1.776 (1.457–2.165)	< 0.001
**Heart problems**						
No	6323 (89.0)	336 (5.3)	5987 (94.7)	< 0.001	1.00 (reference)	
Yes	763 (10.7)	72 (9.4)	691 (90.6)		1.807 (1.401–2.330)	< 0.001
Missing data	22 (0.3)					
**Diabetic Status**						
Others	3126 (44.0)	155 (5.0)	2971 (95.0)	0.032	1.00 (reference)	
Prediabetes	3298 (46.4)	207 (6.3)	3091 (93.7)		1.273 (1.034–1.568)	0.023
Diabetes	684 (9.6)	47 (6.9)	637 (93.1)		1.399 (1.009–1.939)	0.044
**Chronic kidney disease**						
No	6644 (93.5)	376 (5.7)	6268 (94.3)	0.098	1.00 (reference)	
Yes	436 (6.1)	33 (7.6)	403 (92.4)		1.345 (0.942–1.920)	0.102
Missing data	28 (0.4)					
**CES-D scores, median (IQR)**	7 (3, 12)	8.0 (4.0, 14.0)	7.0 (3.0, 12.0)	0.005	1.024 (1.009–1.039)	0.001

*Abbreviation* SD Standard deviation, *IQR* Interquartile range, *hs-CRP* High sensitivity C-reactive protein, *FBG* Fasting blood glucose, *HDL-c* HDL cholesterol, *LDL-c* LDL cholesterol, *BMI* Body mass index, *CESD-10* The 10-item Center for Epidemiology Scale for Depression (CESD-10), *HR* Hazard ratio, *CI* Confidence interval

^#^ The Chi-square test for categorical variables and the t-test or Wilcoxon test for continuous variables were used to assess the differences between the groups

### Independent associations of multiple chronic disease status and depressive symptoms as well as their combination with incident stroke

**Fig. 2:**
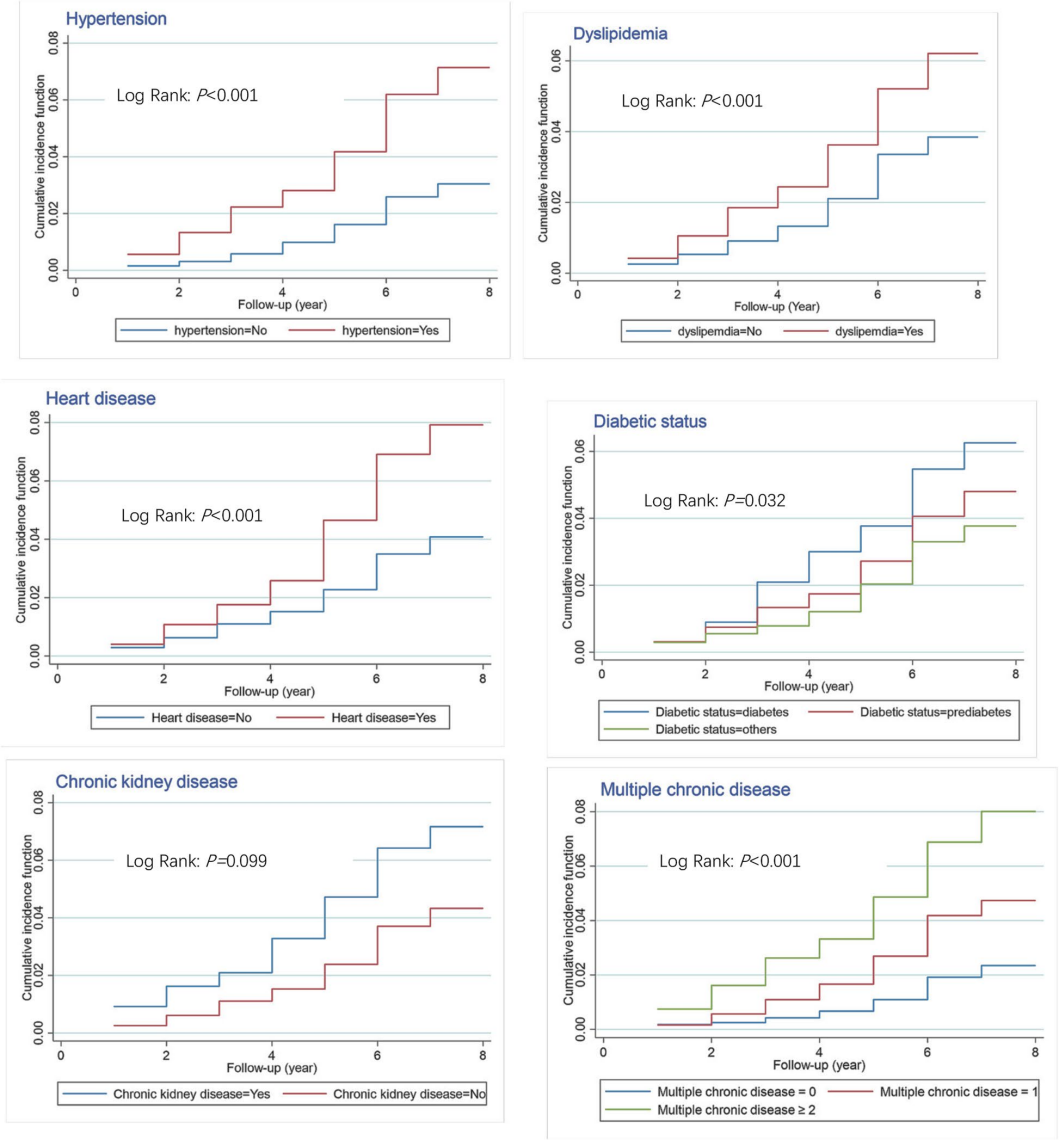
The Nelson-Aalen curves illustrating the occurrence of incident stroke and chronic disease over time

Table 3 displays that the incidence rate of stroke was 4.07 per 1000 person-years among participants without chronic disease, was 7.96 per 1000 person-years among those with 1 type of chronic disease, and was 13.82 per 1000 person-years among those with at least two types of chronic diseases. Besides, the incidence rate of stroke among patients with depressive symptoms was higher (9.16 per 1000 person-years) than those without depressive symptoms (6.97 per 1000 person-years). The unadjusted Nelson-Aalen curves with log-rank tests demonstrating the cumulative hazards of incident stroke by hypertension, dyslipidemia, heart problems, diabetic status, chronic kidney disease, and multiple chronic disease status were shown in Fig. 2, respectively. Without adjusting for other variables, having 1 type or at least 2 types of chronic diseases and having depressive symptoms were positively associated with incident stroke. After adjusting for potential control variables (including depressive symptoms and clinical variables in model 3), individuals with 1 type of chronic disease had a higher risk of incident stroke (Adjusted HR [AHR] = 1.562, 95% CI = 1.124–2.170) than those without chronic disease, and so did the patient with at least 2 types of chronic diseases (AHR = 2.436, 95% CI = 1.702–3.487). Besides, this study also tested the combined association of multiple chronic diseases and depressive symptoms with incident stroke. Compared to those without chronic disease and depressive symptoms, patients having 1 type of chronic disease and depressive symptoms were at 1.943 (95% CI = 1.166–3.238) times higher risk of incident stroke, and those having at least 2 types of chronic diseases and depressive symptoms were at 3.000 (95% CI = 1.846–4.877) higher risk of incident stroke; the magnitudes of the HRs increased by the numbers of having chronic diseases and depressive symptoms.

**Table 3:** The independent associations of multiple chronic disease status and depressive symptoms as well as their combination with incident stroke

	**Incident stroke (case, No.)**	**Incidence rate, per 1000 person-years**	**Model 1**		**Model 2**		**Model 3**	
			**HR (95% CI)**	***P*-value**	**HR (95% CI)**	***P*-value**	**HR (95% CI)**	***P*-value**
**MCD **^**a**^								
0	90	4.00	1.00 (reference)		1.00 (reference)		1.00 (reference)	
1	150	7.71	1.922 (1.480–2.497)	< 0.001	1.607 (1.213–2.130)	0.001	1.562 (1.124–2.170)	0.008
≥ 2	168	13.47	3.336 (2.582–4.309)	< 0.001	2.578 (1.946–3.415)	< 0.001	2.436 (1.702–3.487)	< 0.001
**Depressive symptoms**								
No	294	6.97	1.00 (reference)		NA		NA	
Yes	115	9.16	1.313 (1.058 ~ 1.629)	0.013	NA		NA	
**Combined effects**								
MCD(0)-not having depressive symptoms	67	3.75	1.00 (reference)		1.00 (reference)		1.00 (reference)	
MCD(0)-having depressive symptoms	23	4.97	1.326 (0.825–2.129)	0.243	1.469 (0.897–2.406)	0.127	1.662 (0.953–2.897)	0.073
MCD(1)-not having depressive symptoms	113	7.52	2.002 (1.480–2.708)	< 0.001	1.756 (1.268–2.432)	0.001	1.738 (1.182–2.555)	0.005
MCD(1)-having depressive symptoms	37	8.35	2.219 (1.485–3.315)	< 0.001	1.795 (1.143–2.817)	0.011	1.943 (1.166–3.238)	0.011
MCD(≥ 2)-not having depressive symptoms	113	12.45	3.290 (2.432–4.451)	< 0.001	2.730 (1.963–3.796)	< 0.001	2.793 (1.842–4.234)	< 0.001
MCD(≥ 2)-having depressive symptoms	55	16.23	4.276 (2.993–6.109)	< 0.001	3.262 (2.183–4.876)	< 0.001	3.000 (1.846–4.877)	< 0.001
**Multiplicative interaction**								
Multiple chronic disease × Depressive symptoms	NA	NA	NA	0.941	NA	0.640	NA	0.258

*Abbreviation*: *MCD* Multiple chronic diseases, *No.* Number, *HR* Hazard ratio, *CI* Confidence interval, *NA* Not applicable

Model 1: Unadjusted model

Model 2: Adjusting for gender, age, marital status, education level, ever smoking, last year drinking, residence, and body mass index at baseline

Model 3: Adjusting for the variables in Model 2 plus clinical variables, including fasting blood glucose, triglycerides, creatine, total Cholesterol, HDL cholesterol, LDL cholesterol, High sensitivity C-reactive protein, hemoglobin, uric acid, and Cystatin C

### Sensitivity analyses

Table 4 also shows the results of sensitivity analyses incorporating all types of chronic disease. Without adjusting for other variables (in model 1), among the five types of chronic disease, hypertension (AHR = 2.232, 95% CI = 1.826–2.728), dyslipidemia (AHR = 1.508, 95% CI = 1.228–1.852), and heart disease (AHR = 1.437, 95% CI = 1.107–1.864) were associated with elevated risks of incident stroke. After adjusting for control variables (in model 3), only hypertension (AHR = 1.786, 95% CI = 1.384–2.306) and dyslipidemia (AHR = 1.381, 95% CI = 1.003–1.901) were positively associated with incident stroke, and the magnitudes of the associations for hypertension and incident stroke were most significant.

**Table 4:** Sensitivity analyses of the associations between multiple chronic disease status and incident stroke

**Type of chronic disease**^**a**^	**Incident stroke (case, No.)**	**Incidence rate, per 1000 person-years**	**Model 1**		**Model 2**		**Model 3**	
			**HR (95% CI)**	***P*-value**	**HR (95% CI)**	***P*-value**	**HR (95% CI)**	***P*-value**
**Hypertension**								
No	174	4.90	1.00 (reference)		1.00 (reference)		1.00 (reference)	
Yes	235	12.20	2.232 (1.826–2.728)	< 0.001	1.931 (1.547–2.411)	< 0.001	1.786 (1.384–2.306)	< 0.001
**Dyslipidemia**								
No	246	6.16	1.00 (reference)		1.00 (reference)		1.00 (reference)	
Yes	163	11.00	1.508 (1.228–1.852)	< 0.001	1.453 (1.162–1.818)	0.001	1.381 (1.003–1.901)	0.048
**Heart disease**								
No	336	6.88	1.00 (reference)		1.00 (reference)		1.00 (reference)	
Yes	72	12.50	1.437 (1.107–1.864)	0.006	1.320 (0.988–1.764)	0.060	1.250 (0.896–1.744)	0.189
**Diabetic status**								
Others	155	6.41	1.00 (reference)		1.00 (reference)		1.00 (reference)	
Prediabetes	207	8.17	0.915 (0.655–1.279)	0.603	1.030 (0.714–1.487)	0.874	1.046 (0.588–1.863)	0.877
Diabetes	47	8.98	1.024 (0.744–1.409)	0.883	1.146 (0.808–1.625)	0.446	1.176 (0.717–1.930)	0.520
**Chronic kidney disease**								
No	376	7.34	1.00 (reference)		1.00 (reference)		1.00 (reference)	
Yes	33	9.88	1.251 (0.873–1.791)	0.222	1.243 (0.840–1.838)	0.277	1.353 (0.880–2.079)	0.168

*Abbreviation*: *No.* Number, *HR* Hazard ratio, *CI* Confidence interval, *NA* Not applicable

^a^ Incorporating all the four types of chronic disease in the Cox proportional hazards models

Model 1: Unadjusted model

Model 2: Adjusting for gender, age, marital status, education level, ever smoking, last year drinking, residence, body mass index, and depressive symptoms at baseline

Model 3: Adjusting for the variables in Model 2 plus clinical variables, including fasting blood glucose, triglycerides, creatine, total Cholesterol, HDL cholesterol, LDL cholesterol, High sensitivity C-reactive protein, hemoglobin, uric acid, and Cystatin C

## Discussion

This prospective population-based cohort study found significant associations between multiple chronic diseases (especially hypertension) and incident stroke among middle-aged and elderly adults in China with over 8 years of follow-up, and depressive symptoms played additive effects in the associations mentioned above. Approximately 18.7% of the participants experienced elevated depressive symptoms at baseline, and participants with the presence of depressive symptoms are more possibly to be female, older, separated or divorced, have lower education level, live in rural areas, and have a history of chronic disease than those without depressive symptoms. These findings were consistent with prior studies [14, 16], suggesting that depressive symptoms have been a common public health issue among Chinese adults, and individuals with the aforementioned negative characteristics were more likely to be involved in depressive symptoms.

This eight-year follow-up study observed that the incidence of stroke among Chinese adults aged 45 years or older was 7.47 per 1000 person-years, and this finding was consistent with the 2019 China Stroke Statistics, which indicated that the incidence rates of stroke per 1000 person-years among Chinese adults aged 50–59 years, 60–69 years, and 70–79 years were 4.331, 8.214, and 13.499, respectively [15]. In line with previous evidence [5, 12, 15, 41, 42], this longitudinal study observed that without adjusting for other variables, conventional risk factors including older age, widowed, BMI, systolic pressure, diastolic pressure, depressive symptoms scores, having a history of hypertension/dyslipidemia/heart problems/prediabetes and diabetes were associated with increased risks of incident stroke. However, the univariable analyses in this study did not show a significant association between chronic kidney disease and incident stroke. Although the Reasons for Geographic and Racial Differences (REGARDS) study also reported that chronic kidney disease (defined by eGFR < 60 mL/min/1.73 m^2^) was not associated with stroke risk, this study reported that this finding should be interpreted with caution, considering there were relatively few stroke events among participants with chronic kidney disease limiting the statistical power to detect a more modest association of low eGFR with risk of stroke [43]. This explanation might also be appropriate for our no statistically significant findings about the association between chronic kidney disease and incident stroke.

Moreover, we also found that hs-CRP, FBG, serum creatinine, TC, TG, LDL-c, HDL-c, hemoglobin, uric acid, and cystatin C were associated with incident stroke, with higher levels of the above biochemical parameters (except for HDL-c) being risk factors for incident stroke. As depressive symptoms have been shown to be associated with incident stroke [16, 18], in our univariable analyses, significant associations of depressive symptoms with an increased risk of incident stroke were also observed. Therefore, the covariate effects of some above-mentioned factors should be considered when investigating the associations of multiple chronic diseases with incident stroke. Besides, these findings are helpful in identifying a profile of individuals with a high risk of incident stroke, and community-based programs might be efficient when detecting these people’s profiles.

Moreover, the hypothesis that more chronic diseases were associated with a greater risk for incident stroke than a single chronic disease alone was successfully assessed. This study found that even after adjusting for potential confounders, depressive symptoms, and significant clinical and biochemical indexes, patients having 1 type of chronic disease were at 1.562 times risk of incident stroke than those without chronic disease, while patients having at least 2 types of chronic diseases were at 2.436 times risk of incident stroke at 8-year follow-up. These results might be related to the fact that the common chronic diseases, including hypertension, dyslipidemia, heart disease, and diabetes, were positively associated with the incident stroke [5], and the presence of two or more chronic diseases is a norm among older people and highly prevalent in patients with incident stroke [44]. Having more chronic diseases might mean that patients have poor physical conditions, less health care, less health management skills, or poor family or social support [45]. Furthermore, chronic disease (especially multiple chronic diseases) significantly influences the risk of incident stroke and may even worsen the subsequent quality of life, health status and recovery, and mortality [46]. We recommended developing action planning, interactive learning, and behavior modeling to help patients with chronic disease and their families build health management skills and coping strategies to address these problems and reduce the risk of stroke or other adverse health conditions.

Moreover, given that patients with multiple chronic diseases may also experience a great deal of suffering from depressive symptoms, our study also examined the combined effects of multiple chronic diseases and depressive symptoms on incident stroke. We observed that compared with those without chronic disease and depressive symptoms, patients with the presence of 1 type of chronic disease and depressive symptoms were at 1.943 times higher risk of incident stroke, and those with at least 2 types of chronic disease and depressive symptoms were at 3.000 times higher risk of incident stroke. There was a dose–response association of the numbers of chronic disease and depressive symptoms with incident stroke. However, few previous studies analyzed the combined effects of chronic physical disease and depressive symptoms on the risk of incident stroke. A possible explanation for our findings might be that both chronic disease and depressive symptoms have been identified to be positively associated with the incident stroke in this study and previous evidence [8, 16]. Most middle-aged and older adults with chronic diseases were more likely to develop depressive symptoms, and patients experiencing depressive symptoms also increased the risks of the presence of multiple chronic diseases and the number of comorbidities [47]. Although this study did not find significant multiplicative interactions between chronic disease and depressive symptoms on incident stroke, another explanation for the combined effects may be that multiple chronic conditions may combine with depressive symptoms to accelerate the activation of the inflammation, endothelial dysfunction, hypothalamic-pituitary-adrenocortical axis or autonomic nerve dysfunction and subsequently affect the biological process of stroke [48, 49].

In addition, the sensitivity analyses of this study also observed that hypertension is a major contributor to incident stroke among Chinese middle-aged and older adults, followed by dyslipidemia. Similarly, previous evidence also strengthened that the leading risk factor for stroke was hypertension, and improving hypertension awareness, treatment, and control was highly recommended [15]. Nevertheless, the management of other chronic diseases to reduce stroke risk should not be ignored, considering the comorbidity between chronic disease and mental health problems.

The strengths of the current study include the large-scale, 8-year longitudinal follow-up study design, the use of incident stroke as an outcome, and the use of extensive information collected from questionnaires, physical examination (i.e., BMI and blood pressure), and clinical and biochemical measurements (such as FBG, HbA_1c_, hs-CRP, creatine, and hemoglobin) to unbiased estimate the independent and combined associations of multiple chronic disease and depressive symptoms with incident stroke.

Our study also had several limitations. First, the study sample only included Chinese, and then the generalization of the findings may not be applicable to other ethnicities. Future research is needed in more diverse groups. Second, we did not distinguish incident stroke in terms of type, but most strokes are ischemic strokes, and the hazard of chronic disease on the different types of stroke is similar. Third, although this study utilized an eight-year longitudinal study design, associations still should be cautiously interpreted because they were generated from an interval that might not be long enough to uncover incident stroke. Fourth, although some information collected through the standardized questionnaire (e.g., the variable of incident stroke and chronic diseases) was measured by self-reported, which cannot avoid self-report bias, self-reports remain a common and accepted method [36]. Fifth, there may be over-adjustment bias, as several control variables are included in the adjusted models based on the univariable analyses results and knowledge obtained from the literature [37–40].

## Conclusions

In summary, this population-based longitudinal study identified the significant independent and combined longitudinal associations of multiple chronic diseases and depressive symptoms with incident stroke. Although the multiplicative interaction between chronic disease and depressive symptoms on incident stroke was not observed, the combined associations reflected a dose–response relationship on incident stroke, with the numbers of chronic disease and depressive symptoms increasing. Besides, the association between hypertension and incident stroke was strongest among the five types of chronic diseases. Improving the management of chronic conditions and depressive symptoms may reduce the risks of incident stroke among Chinese middle-aged and older adults.

## Supplementary Information

**Additional file 1: Supplementary Figure S1.** Timeline and procedures of the China Health and Retirement Longitudinal Study. Multiple chronic disease and depressive symptoms were explored at baseline, and incident stroke was followed biennially for up to 8 years.

## Data Availability

The data that support the findings of the study are available through the website of the CHARLS: http://charls.pku.edu.cn/index/en.html. To access and use the day for research purpose, approval should be obtained from the CHARLS team at Peking University.

## Abbreviations

DALY: Disability-adjusted life-years
hs-CRP: High-sensitivity C-Reactive Protein
CHARLS: China Health and Retirement Longitudinal Study
CESD-10: 10-Item Center for Epidemiology Scale for Depression
BMI: Body mass index
CDC: Center of Disease Control
FBG: Fasting blood glucose
HPLC: High-performance liquid chromatography
TC: Total cholesterol
HDL-c: HDL cholesterol
LDL-c: LDL cholesterol
TG: Triglyceride
ADA: American Diabetes Association
IQR: Interquartile range
HRs: Hazard ratios
Cis: Confidence intervals
