# Supplementary material for: Associations of multiple chronic disease and depressive symptoms with incident stroke among Chinese middle-aged and elderly adults: a nationwide population-based cohort study
Source: BMC Geriatr. 2022 Aug 12;22:660. doi: 10.1186/s12877-022-03329-4 (PMC9373457; doi:10.1186/s12877-022-03329-4)
Supplement: Supplementary file 1 [file 12877_2022_3329_MOESM1_ESM.docx]

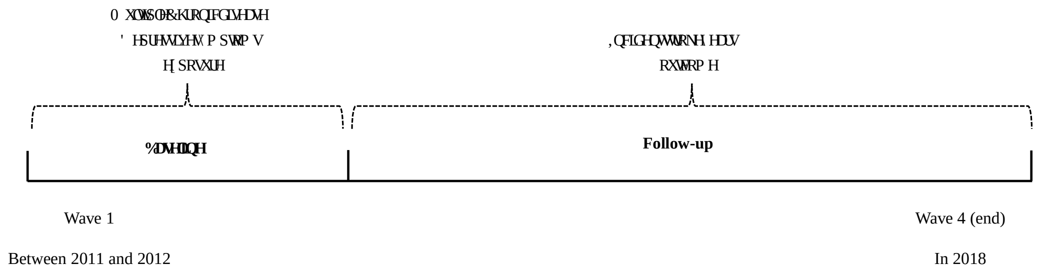

Supplementary Figure S1. Timeline and procedures of the China Health and Retirement Longitudinal Study. Multiple chronic disease and depressive symptom were explored at baseline, and incident stroke was followed biennially for up to 8 years.
